# SeamDock: An Interactive and Collaborative Online Docking Resource to Assist Small Compound Molecular Docking

**DOI:** 10.3389/fmolb.2021.716466

**Published:** 2021-09-17

**Authors:** Samuel Murail, Sjoerd J. de Vries, Julien Rey, Gautier Moroy, Pierre Tufféry

**Affiliations:** ^1^ CNRS UMR 8251, INSERM ERL U1133, Université de Paris, Paris, France; ^2^ Ressource Parisienne en Bioinformatique Structurale (RPBS), Paris, France

**Keywords:** molecular docking, web-server, virtual screening, drug discovery, collaborative sessions

## Abstract

*In silico* assessment of protein receptor interactions with small ligands is now part of the standard pipeline for drug discovery, and numerous tools and protocols have been developed for this purpose. With the SeamDock web server, we propose a new approach to facilitate access to small molecule docking for nonspecialists, including students. The SeamDock online service integrates different docking tools in a common framework that allows ligand global and/or local docking and a hierarchical approach combining the two for easy interaction site identification. This service does not require advanced computer knowledge, and it works without the installation of any programs with the exception of a common web browser. The use of the Seamless framework linking the RPBS calculation server to the user’s browser allows the user to navigate smoothly and interactively on the SeamDock web page. A major effort has been put into the 3D visualization of ligand, receptor, and docking poses and their interactions with the receptor. The advanced visualization features combined with the seamless library allow a user to share with an unlimited number of collaborators, a docking session, and its full visualization states. As a result, SeamDock can be seen as a free, simple, didactic, evolving online docking resource best suited for education and training.

## 1 Introduction

Molecular docking is a computational tool that attempts to predict the structure of interaction between a protein and a molecule. Roughly, docking programs are a combination of a search algorithm and a scoring function. The search algorithm aims to find the precise ligand 3D geometry, also called poses, within a given targeted protein. The scoring function purpose is the prediction of the binding affinity in order to evaluate how well the ligands bind to the protein. The molecular docking of small molecules to protein binding sites was pioneered during the early 1980s (Kuntz et al., 1982). In the past 40 years, a large set of different methods and programs have been developed and enhanced to provide more and more suitable predictions. In parallel, docking developments have been proposed to address more specific questions such as the search for protein–protein interaction (PPI) inhibitors (Ran and Gestwicki, 2018), allosteric drugs (Wagner et al., 2020), or fragment-based approaches (Shan et al., 2020). Nowadays, molecular docking protocols are widely used to develop new drugs and are an important part of the drug discovery pipeline at pharmaceutical companies (Muegge et al., 2017). Docking protocols, include in their pipeline, the use of docking software such as DOCK 6 (Lang et al., 2009), AutoDock 4 (Morris et al., 2009), EADock (Grosdidier et al., 2007), AutoDock Vina (Trott and Olson, 2010), and rDock (Ruiz-Carmona et al., 2014). The following non-exhaustive list presents standalone programs that require a minimal computing and biophysics knowledge to be used correctly.

Important progress has been made in computer science to simplify and facilitate the installation of programs and libraries; we can mention some package managers such as apt-get, brew, pip, or conda. However, a user who is not familiar with Unix command lines may find the installation and use of docking programs such as AutoDock, AutoDock Vina, and MGLTools (Morris et al., 2009) or visualization programs such as VMD (Humphrey et al., 1996), PyMOL (The PyMOL Molecular Graphics System, Delano Scientific, San Carlos, CA, USA), or Chimera (Pettersen et al., 2004) too complex. Moreover, the limitations of access and writing permissions on particular computer equipment make the installation of these programs even more complex and laborious. This is particularly the case in a pedagogical context (e.g., practical work), where the use of different Unix command line programs leads to a waste of time for students and supervisors. In our experience, most of the student issues come from a bad understanding of the command line environment. In the worst case, this distracts the students from the educational purpose of the session.

To improve the accessibility of such computational resources, some of the previously cited *in silico* approaches have been made accessible online thought web servers. Examples of web services for small molecule docking are as follows: Webina (Kochnev et al., 2020) which allows the user to run AutoDock Vina; SwissDock (Grosdidier et al., 2011b) a web interface based on EADock docking software (Grosdidier et al., 2011a); DockThor (Guedes et al., 2021) a web server focused on SARS-CoV-2 therapeutic targets; and MedusaDock (Wang and Dokholyan, 2019) a docking methodology capable of incorporating structural constraints.

Although well-adapted for research, current online docking facilities have generally one or more limitations, such as absence of collaborative working, limited interactivity (definition of the grid, visualization, and graphical analysis of the results), and navigation through multiple submission pages. Particularly, in the context of education, group sessions using the same data usually imply a duplication of the runs, and sharing result analyses interactively is not possible.

With the SeamDock web server, we propose a new approach to facilitate access to small-molecule docking for nonspecialists, including students. SeamDock can be seen as interfacing a docking library interactively through a web browser. Presently, the docking library embeds an interface with several docking engines, and interactive visualization is ensured using the NGL viewer (Rose et al., 2018). The design of the interactive web interface relies on the open source Seamless framework (https://github.com/sjdv1982/seamless) that brings innovation in terms of reproducibility, interactivity, and sharing. Each SeamDock run is in fact an interactive docking session. Communication between the browser and server is in real-time and bidirectional. Thus, there is no global submit button: the SeamDock server is informed gradually of changes in the docking inputs. Likewise, progress, results, and error messages from the server are continuously displayed in the main web page.

In addition, docking sessions are collaborative. Each session has a persistent URL, and multiple users can connect to this URL in their browser. Using Seamless, session state is synchronized in the browser: each user can modify the docking inputs and will be notified of all changes. Seamless synchronization is not limited to inputs and docking results alone. In SeamDock, visualization parameters such as camera orientations are also synchronized. The simple sharing of docking sessions over many browser instances makes it remarkably suitable for teaching to a large audience. As a result, SeamDock can be seen as a simple, didactic, evolving online docking resource best suited for education and training. The SeamDock web site is available online at https://bioserv.rpbs.univ-paris-diderot.fr/services/SeamDock/.

## 2 Materials and Methods

**FIGURE 1:**
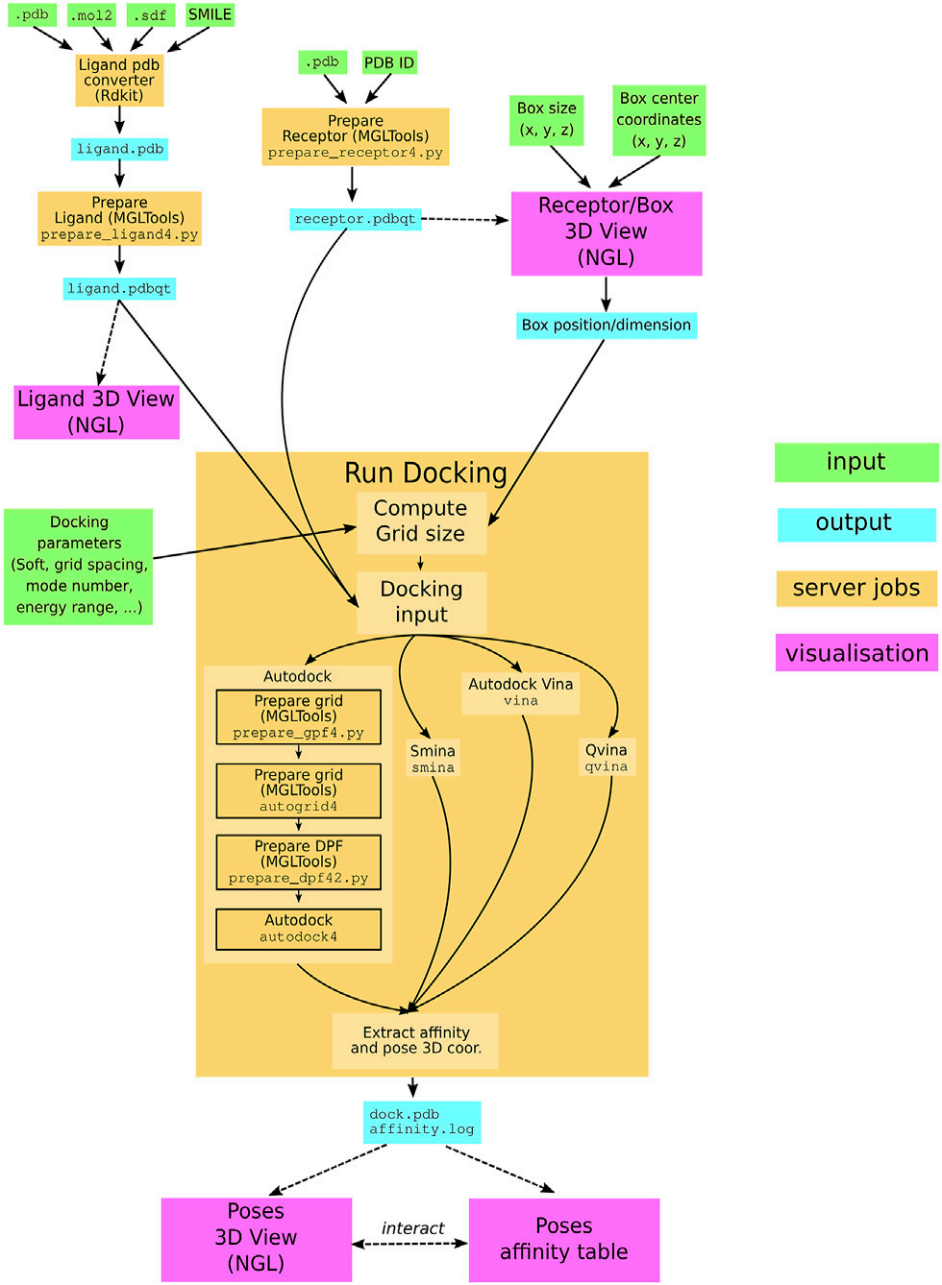
SeamDock Workflow. Schematic flow diagram, showing the general pipeline of SeamDock. The user enters input, shown as green boxes, using the SeamDock web page. The user inputs are synchronized with the web server using JavaScript and Seamless. On the server, inputs, jobs, and outputs are defined in a Seamless workflow. Seamless will execute jobs upon input changes. Jobs are implemented as Python scripts using docking_py, invoking the various docking tools. Results are synchronized with the web page using Seamless. Output structures are then shown using tables and the NGL viewer.

The SeamDock workflow (Figure 1) involves on the server side the use of the docking_py library for ligand/receptor preparation and docking computation, as on the web side, JavaScript in combination with the NGL viewer (Rose et al., 2018) allows for a full visualization of ligand and the receptor structure as well as docking poses and their interaction with the receptor. Communication between the web page and the server is handled by the Seamless framework, which updates the workflow in response to changes in the web page inputs.

### 2.1 Docking_py Library

The SeamDock application run on the server side through the in house developed Python library docking_py (Tufféry and Murail, 2020). Docking_py is a python library allowing a simple and unified use of the docking software AutoDock 4 (Morris et al., 2009), AutoDock Vina (Trott and Olson, 2010), Qvina (Hassan et al., 2017), and Smina (Koes et al., 2013). Docking_py is an open-source library which code is versioned and deposited on the GitHub platform at https://github.com/samuelmurail/docking_py. The code development takes advantage of continuous integration through the Travis services (https://travis-ci.org), allowing a code testing at each code modification. Code documentation is available at https://docking-py.readthedocs.io. For simple user installation, the code has been deposited on the Bioconda channel (https://bioconda.github.io/recipes/docking_py/README.html) and on the Pypi repository (https://pypi.org/project/docking-py/). The docking_py library has been optimized to be used in Jupyter notebooks, with 3D docking results being displayed with the help of nglview (Nguyen et al., 2018) library (see as example https://docking-py.readthedocs.io/en/latest/notebook/Usage.html).

### 2.2 Seamless

The SeamDock web server is implemented with the open source Seamless framework (https://github.com/sjdv1982/seamless). A Seamless workflow is a graph of connected cells that can contain data or code. A cell can be shared and synchronized over HTTP, and mounted to the local file system. Cells can contain scientific data, code, results, or web content (html, css, js). Between cells, transformations can be defined that connect a code cell and input cells to a cell that will contain the result. The code in a code cell can be written in any programming language: currently, Seamless supports Python, bash, Cython, Fortran, C, and C++. Transformations may be run in a Docker container. For SeamDock, transformations are forwarded as Singularity jobs to our HPC cluster. Seamless describes cell contents as checksums, not values. Likewise, transformations are described in terms of checksums, not file names or URLs. Transformation result checksums are remembered indefinitely, which means that if a transformation has ever been performed before, it is completed instantly. Seamless graphs are executed continuously, and cells can be changed, added and removed on the fly. Seamless makes no hard distinction between developers (who can add cells and change code cells) and users (who can change cell values using a web form).

### 2.3 Input Formatting

Ligand input follow a two-step preparation, in a first step, the ligand is converted to a pdb format using the RDKit (http://www.rdkit.org) open-source chemoinformatics library. Currently, the ligand converter accepts mol2, sdf, and SMILES formats. To compute and optimize the 3D structure of ligand structure starting from a SMILES 1D or sdf/mol2 2D representation, we use the default parameters of the rdkit function MMFFOptimizeMolecule, with the Merck molecular force field MMFF94 (Halgren, 1996) and a maximum number of iterations of 200. Ligand coordinate center of mass is centered on position (0, 0, 0). The ligand pdb file is then processed using the prepare_ligand4.py from AutoDock Tools (Morris et al., 2009), which assign atom types, compute the atomic charge, and repair hydrogen atoms if missing. All ligand torsions are kept active, with the exception of peptidic ligand for which the backbone dihedral angles will be freeze. The output is a pdbqt file that will be used as input for all docking software.

To prepare the receptor structure, first the receptor center of mass is centered on position (0, 0, 0), and a filter is applied on amino and nucleic acid residues to remove water or ligand from the receptor and keep only protein, DNA, and RNA atoms. The receptor coordinate extensions are extracted and used to define the maximum value of the docking box size. The protein residue protonation is computed with the pdb2pqr python library (Dolinsky et al., 2007), using the Propka method (Olsson et al., 2011) at a pH of 7.0. The prepare_receptor4.py script from AutoDock Tools (Morris et al., 2009) is then executed to compute the atom types and charges of the receptor and to add hydrogen atoms if missing.

### 2.4 Docking Procedure

Once the user has defined a ligand structure, a receptor structure, the docking box, and the docking parameters, the user can launch the docking using the dedicated web form button. The docking procedure differs depending on the selected docking software. For AutoDock docking, two successive steps are executed to compute the docking grid (using MGLTools preprare_gpf4.py to prepare the grid parameter file and autogrid4 to compute the grid). The prepare_dpf4.py from MGLTools is then launched to prepare the docking parameter file, which is then used as input for autodock4. For Vina, Smina, and Qvina, the procedure is simple, as no grid or input parameter file needs to be precomputed. The three docking software are directly launched with the receptor and ligand pdbqt files defined as input.

### 2.5 Input/Output Visualization

To allow the user an interactive and complete 3D visualization of the structures of the different molecules in play, we have fully integrated the 3D viewer NGL Viewer (Rose et al., 2018) in the web page. For both ligand and receptor input, a 3D visualization stage allows the user to inspect the computed 3D structures. Similarly the different docking pose 3D structures can be inspected interactively. NGL Viewer is a key feature of SeamDock, as it allows the user to interactively visualize and modify the docking box over the receptor structure. Moreover NGL viewer is used in the docking outputs to compute all ligand–receptor interactions. JavaScript functions allow to display interactively, in the NGL stage, selected docking pose from the results table, or to highlight a molecular contact from the interaction tables.

## 3 Results

Users can easily proceed to a molecular docking without extensive computing or biophysics knowledge. Our web server does not necessitate any software installation but only a simple web browser. The user needs to provide a ligand and a receptor file or ID. The user can then choose among the four proposed docking software; AutoDock 4 (Morris et al., 2009), Vina (Trott and Olson, 2010), Qvina (Hassan et al., 2017), and Smina (Koes et al., 2013), and can choose over a limited number of docking options. After few minutes, docking results will be updated on the user web page, giving access to a full 3D molecular visualization of docking poses and their computed affinity.

### 3.1 Ligand Input

**FIGURE 2:**
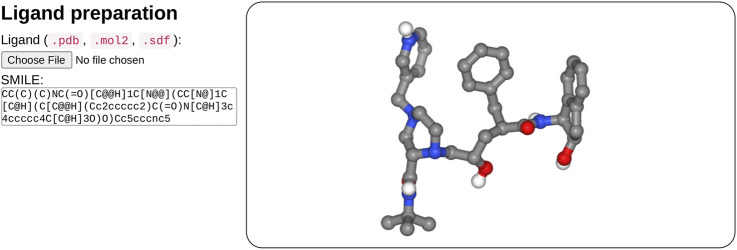
Overview of the ligand input interface. SeamDock provides different options for ligand input. Users can provide ligand files of type pdb, mol2, or sdf, a SMILES specification can also be provided. Once the ligand structure has been prepared, its 3D structure will be displayed in a NGL stage.

In the ligand input part (see Figure 2), the user can specify the ligand structure either by providing a file in the mol2, sdf, or PDB format or enter a SMILES in the text field. One great advantage of the SMILES format is that most chemical databases provide compound description in this format, which can further be edited interactively by the user, for example, to add or change a chemical group, for instance, to demonstrate the impact of a modification on the docking results. Besides, very few web sites make it possible to interactively compute the 3D structure of a compound. Usually, the compound library is preprocessed prior to the calculations (e.g., https://molview.org/).

After input completion (enter or mouse focus change), ligand preparation usually requires few seconds depending on the size and complexity of the ligand. Its structure is then displayed in 3D in a NGL viewer (Rose et al., 2018). Users can thus check that the ligand molecule structure has been correctly generated. Of note, the initial input structure displayed will probably differ from that resulting from the docking since SeamDock performs flexible docking.

### 3.2 Receptor and Docking Box

**FIGURE 3:**
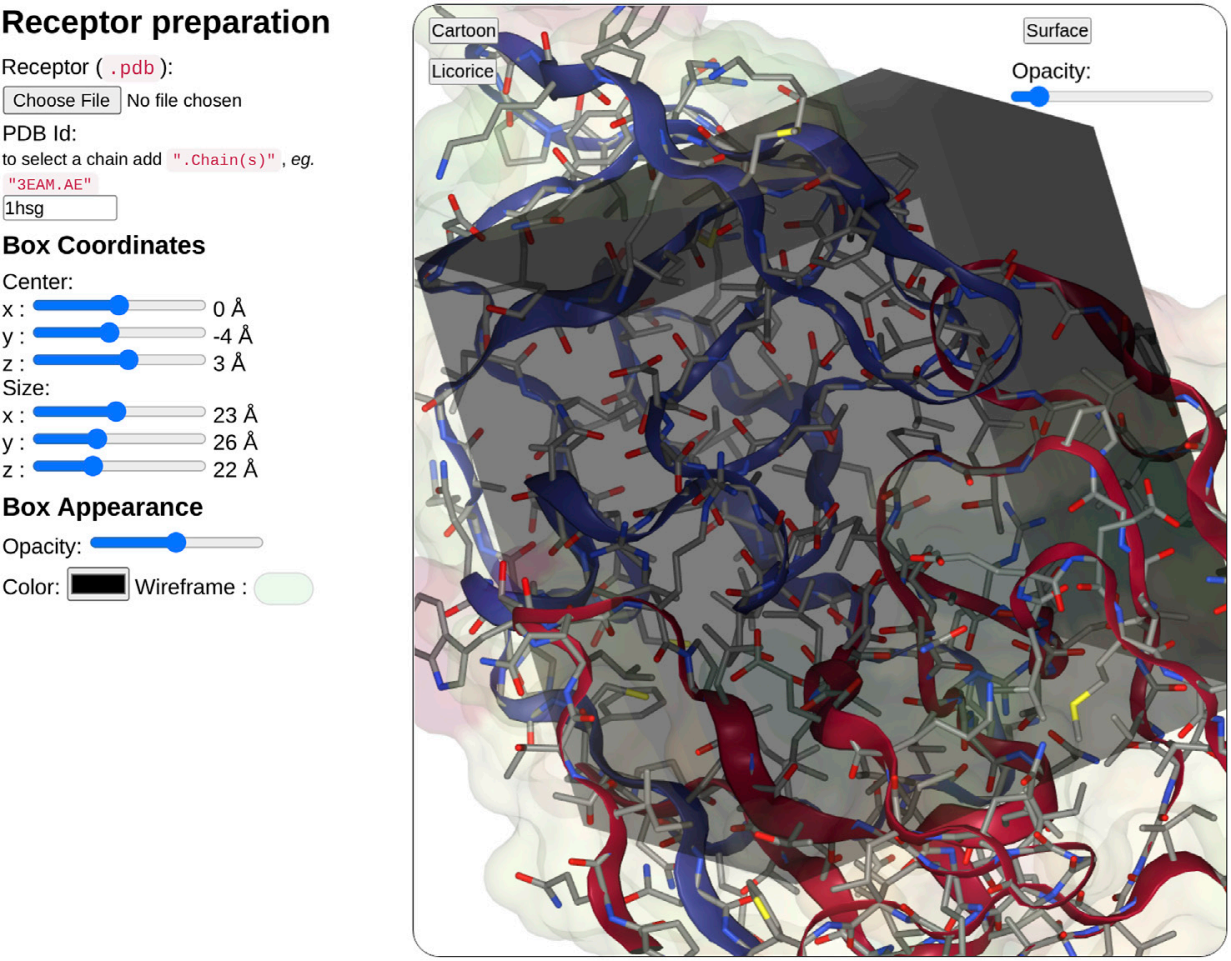
Overview of the receptor input interface. SeamDock provides two options for receptor input. Users can provide a receptor structure file of type pdb, or enter a PDB ID, with or without a selection of chains. After receptor preparation completion, its 3D structure will be displayed in a NGL stage. The NGL stage will also show the docking box, in which the position and size can be adjusted interactively through the six sliders on the left side. Box and receptor appearance can be personalized by the user.

In the receptor input part (see Figure 3), the user can upload a pdb file or enter a PDB ID. When entering the PDB ID, the user can select one or more chains by entering the PDB ID followed by a mark point and the selected chains, for example, to use chains A and E from 3EAM structure enter: “3EAM.AE”. A first filter will extract amino acid and nucleic acid residues to keep only the protein, DNA, and RNA molecules.

Once the receptor structure has been prepared, its structure is displayed in a second instance of NGL viewer. Thanks to NGL, the receptor display can be personalized to show or hide the receptor surface and its opacity, the protein/DNA/RNA secondary structure as a cartoon representation, and the receptor atoms using a stick representation.

Users have then to define the docking box position and dimension. The box definition will restrict the ligand docking process in the specified volume. Users can then define interactively the box position (*x*, *y*, *z*) and size (*x*, *y*, *z*) using six sliders located on the left of the receptor structure. Changing slider values, the NGL viewer will instantly update the box position and size respective to the receptor structure. The box appearance can be personalized with options such as color, opacity, or wire-frame vs. surface representation.

### 3.3 Docking Parameters

For the sake of simplicity, docking parameters are limited to few options. The main option being the docking software (AutoDock, AutoDock Vina, Smina, or Qvina). For Vina, Smina, and Qvina, the user will have to use a 1.0 Å spacing, as with AutoDock, the user can specify it (default value: 0.375 Å). The user can then define the *mode number* and the *energy range* in *kcal*.*mol* ^−1^; the *mode number* defines the maximum number of predicted docking to be generated within the defined *energy range*. Poses with affinity not within the energy range to the best pose will be discarded, no matter if the maximum number of docking poses is not reached. At last the user has to define the *exhaustiveness* which corresponds to thoroughness of search and is roughly proportional to time. For Vina, a value of eight is recommended.

### 3.4 Docking Output

**FIGURE 4:**
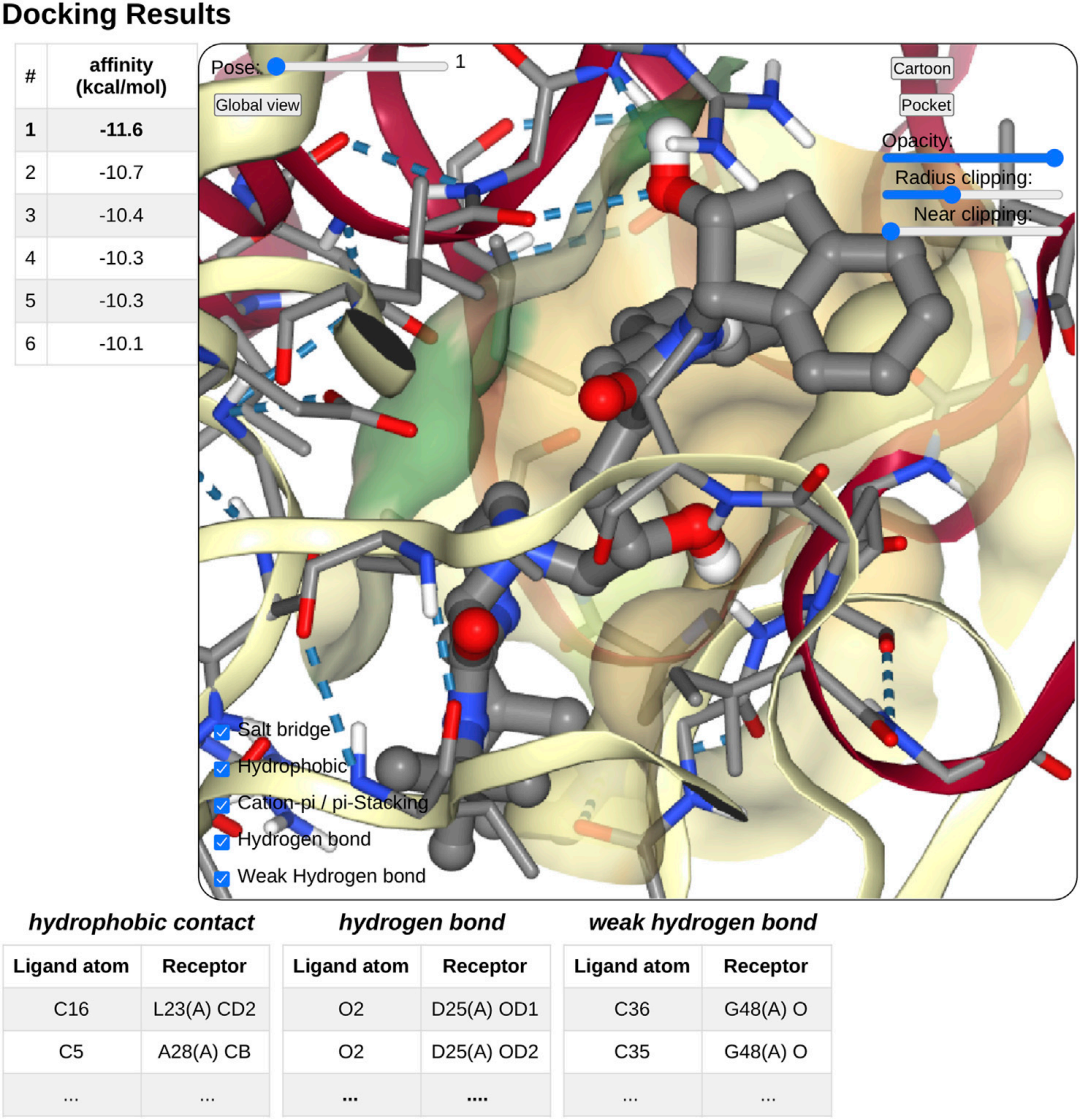
Overview of the docking result interface. Once docking computation has been complete, the docking pose structures in complex with the receptor will be displayed in 3D in a NGL stage. The affinity of each poses will be displayed as a table in a right panel, clicking on a table row will display the selected pose in the NGL stage. Receptor appearance and ligand-receptor interactions can be personalized by the user. Tables of ligand–receptor interactions will be displayed at the bottom of the NGL stage, clicking on a contact will highlight it in the NGL stage.

On docking completion, the identified poses are displayed in a dedicated NGL viewer instance (Rose et al., 2018). Affinity values in *kcal*.*mol* ^−1^ from the docking software are displayed in a table with the pose number (see Figure 4). The table and 3D visualization are synchronized. Clicking on a table row will display the selected pose structure in the NGL stage, as updating the pose slider in the NGL stage will highlight the selected pose in the table.

Pushing the pocket button triggers the display of the binding pocket on the receptor surface. Several options are available to modify the appearance of the surface for clarity, as transparency, radius of extension, and near-clipping. Protein residues within 5.0 Å of the ligand molecule will be displayed as sticks. The protein cartoon representation can be switch on and off.

The NGL viewer allows the user to display different kinds of interactions between the protein and the ligand such as salt bridges, hydrophobic interactions, cation-*π* and *π* stacking, as well as hydrogen bonds. All kinds of interactions can be switched on or off, using a checkbox on the NGL viewer. A table at the bottom of the NGL stage will list in detail the interactions that the user has chosen to display. When updating the docking pose, the new pose interaction table will be updated. The user can highlight a specific interaction by clicking either on the table row of interest or on the contact line in the NGL stage. The contact will be highlighted in magenta in the NGL stage, and the table row text font will be switched to a bold style. By passing the mouse cursor on an atom or a contact, information about the atoms involved (residue number and type, chain, and atom name) will be shown in the NGL scene.

### 3.5 Mastering Display

**FIGURE 5:**
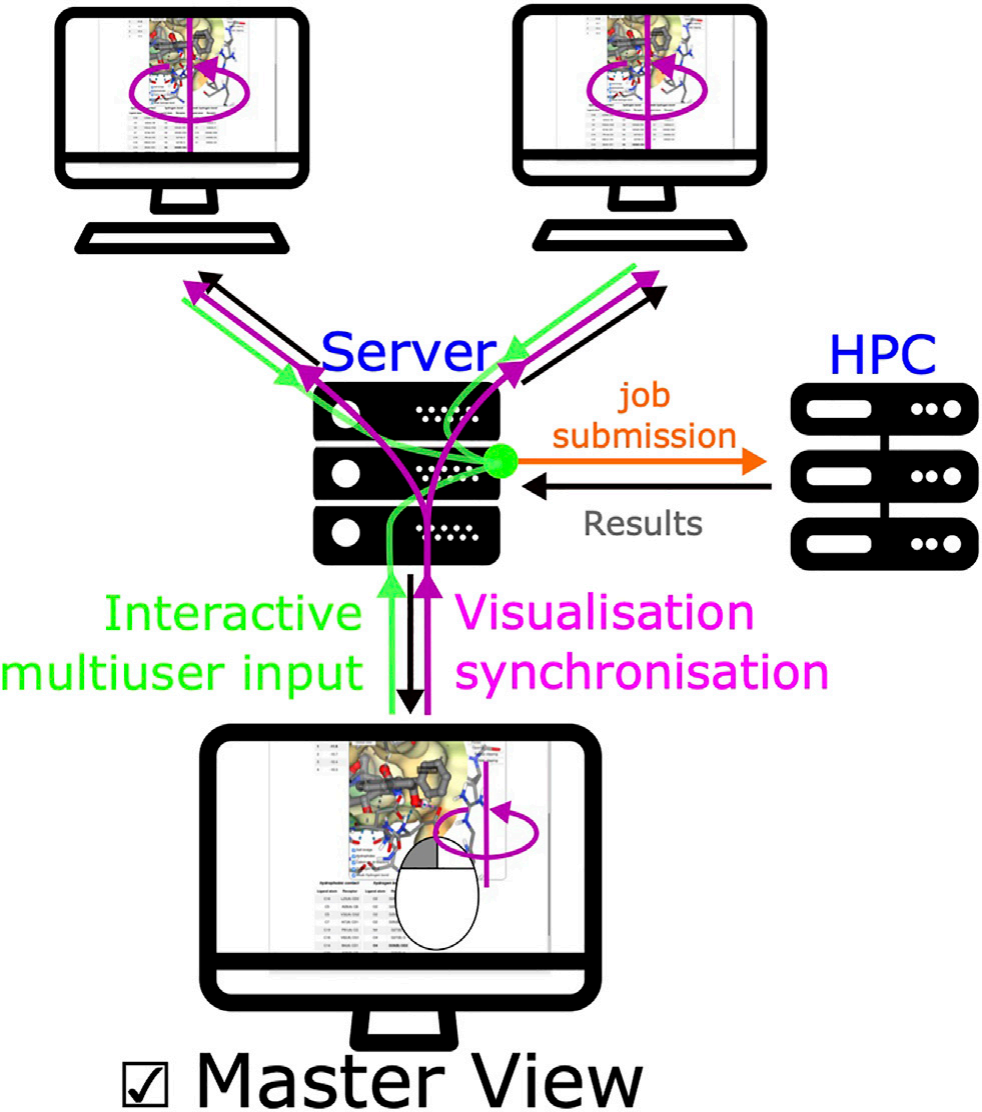
Synchronized view mechanism. The synchronized view mechanism uses the seamless feature for communication between server and client. Once a user ticks the master view button, its web browser will send the NGL stage’s camera orientation and visualization options to the server *via* seamless, other clients’ web browser will then update molecular visualization options. Any modification of input/output (ligand, receptor, or docking results) will be updated on all pages.

A unique feature of SeamDock is the possibility to master the display of the session over a series of browsers, such as in the case of a practical training session. Synchronization between browsers is fully automatic for receptor and ligand coordinates, as well as for all results and error messages of the individual steps of the docking process. In addition, docking parameters are synchronized whenever the “Launch Docking” button is pressed. Finally, a user can become a “master” of the session. With this, the full molecular visualization state (camera orientation, active docking pose, and molecular representations; see Figure 5) is propagated interactively from the master to the server and then synchronized to all viewers accessing the same session. It thus becomes possible to interactively demonstrate pocket definition, important residues, and key interactions, even for users dispersed all over the world.

## 4 Conclusion

The SeamDock web server intends to provide a free and accessible molecular docking tool, in particular for teaching. SeamDock’s ease of use combined with a complete 3D visualization in a collaborative mode makes it a perfect tool for nonspecialists outside of the molecular modeling community. It can be used in a collaborative mode with partners all around the world or in a classroom focused on docking methods or receptor–ligand interactions, while ignoring software installation and Unix command lines. Future developments include an increase in the number of docking engines, a better control of protonation, and a choice for scoring function. In the longer term, integration with experimental constraints used in protein–protein docking protocols such as the HADDOCK web server (van Zundert et al., 2016) could be incorporated, allowing the integration of mass spectrometry cross-linking data or photoaffinity labeling data among others. In addition, integration with third-party web servers could facilitate the selection of binding sites based on ligandability prediction (Jendele et al., 2019).

## Data Availability

The original contributions presented in the study are included in the article; further inquiries can be directed to the corresponding author.
